# Pentoxifylline in COVID-19 and considerations for its research in long COVID

**DOI:** 10.1007/s00011-024-01942-0

**Published:** 2024-10-24

**Authors:** Ahmed Ramzi, Subhia Maya, Nadeen Balousha, Mufreh Amin, Mostafa Ramzi Shiha

**Affiliations:** 1 Faculty of Medicine, Mansoura University, Mansoura, Egypt; 2 Faculty of Medicine, Damascus University, Damascus, Syria; 3 Faculty of Pharmacy, Mansoura University, Mansoura, Egypt; 4 Faculty of Medicine, Misr University for Science and Technology, Giza, Egypt; 5 Faculty of Urban and Regional Planning, Cairo University, Giza, Egypt

**Keywords:** COVID-19, Pentoxifylline, Long COVID, Hospitalization, Systematic Review, Meta-Analysis

## Abstract

**Introduction:**

Pentoxifylline (PTX) affects most blood components and the blood vessels, potentially modulating various conditions. Due to its impact on markers linked to COVID-19 severity, research has explored PTX for acute COVID-19. Following the widespread administration of COVID-19 vaccinations, there has been a notable and consistently growing increase in research focusing on long COVID. Consequently, our examination of relevant acute COVID-19 data shall additionally be contextualized into long COVID research.

**Methods:**

Various Databases were searched until July 2024 for all primary clinical studies on Pentoxifylline (PTX) in COVID-19.

**Results:**

Studies were on acute infection with SARS-CoV-2 where PTX was an adjuvant to standard therapy for ethical and practical reasons under the circumstance. PTX generally reduced hospitalization duration and improved some inflammatory markers, but its impact on mortality was inconsistent. Adverse events were minimal. Meta-analysis revealed a significant reduction in hospitalization duration.

**Conclusion:**

This systematic review and meta-analysis suggest that adding pentoxifylline (PTX) to standard COVID-19 therapy may significantly reduce hospitalization duration and improve some inflammatory markers. However, its impact on mortality rates is inconclusive. Adverse events are minimal. PTX can be favorable as an add-on in managing acute COVID-19 and could reduce the risk of long COVID, as well as assist in managing many of its most common symptoms.

**Supplementary Information:**

The online version contains supplementary material available at 10.1007/s00011-024-01942-0.

## Introduction

Pentoxifylline (PTX), a synthetic methylxanthine introduced in the 1970s, exerts numerous pharmacological effects mainly by inhibiting phosphodiesterase (PDE3 and PDE4) leading to increased cAMP, and acts as an adenosine A2 receptor antagonist [1–5]. It has a short half-life, requiring extended-release formulations and multiple daily doses [6]. PTX has versatile effects on blood components (erythrocytes, leukocytes, platelets, plasma fibrinogen, and inflammatory cytokines such as TNF-α, IL-1, IL-6) and blood vessels [4, 7–12]. Consequently, it could modulate various diseases and conditions [9].

COVID-19 disease severity was linked to multiple hematological markers (lymphopenia, thrombocytopenia, neutrophilia) and inflammatory markers (CRP, IL-6, ferritin) [13–18], providing a rationale for research on PTX in COVID-19, as it affects many of those markers [19–21].

Here we identified those empirical studies and involved them in a systematic review of clinical studies and a meta-analysis, aiming to evaluate the effects of including Pentoxifylline (PTX) in the treatment of acute COVID-19, covering its influence on several clinical outcomes and inflammatory markers, including length of hospital stay, mortality rate, ICU admission, adverse events, CRP, IL-6, and LDH, and could be considered the first one assessing empirical data on the topic.

**Fig. 1:**
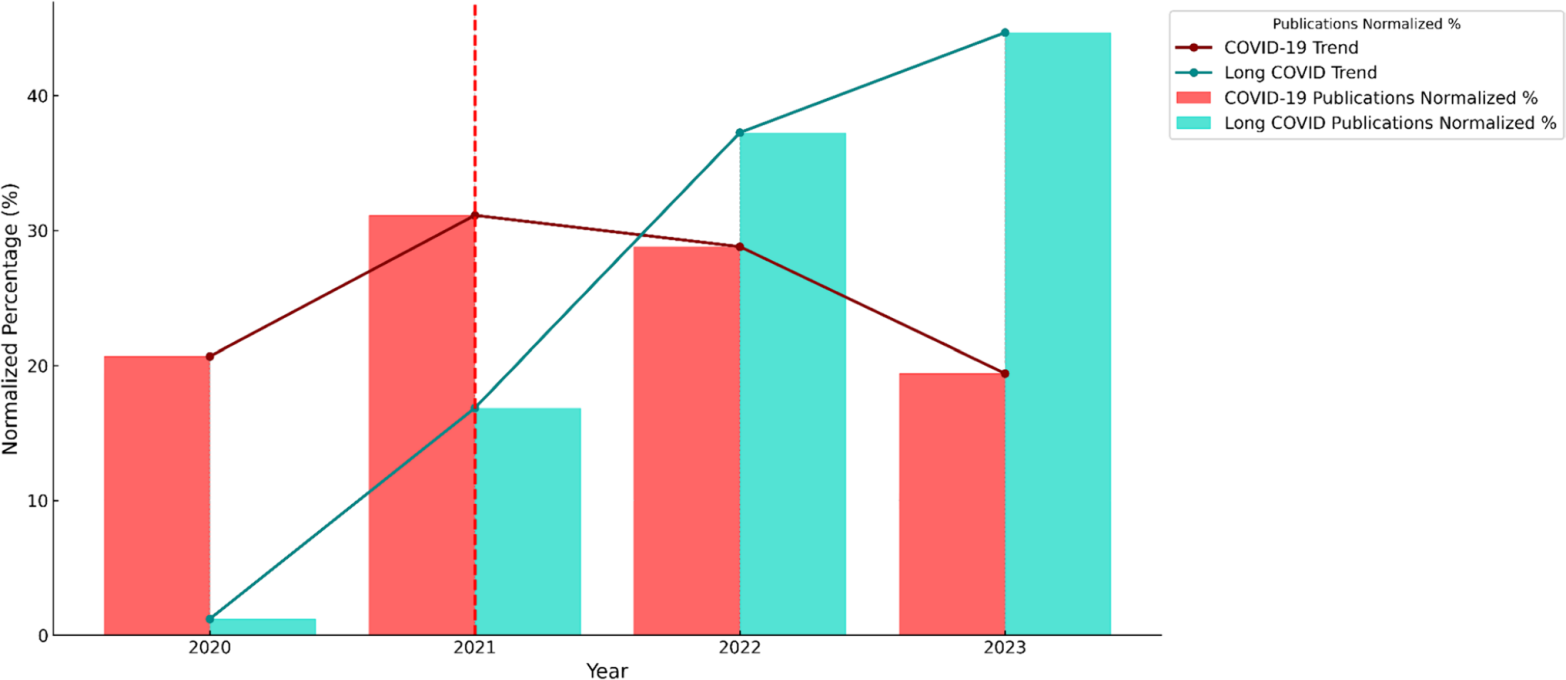
Normalized percentage of publications on COVID-19 (red bars) and Long COVID (turquoise bars) from 2020 to 2023. The trend for COVID-19 publications (red line) peaked in 2021 at 31.12% and declined to 19.42% by 2023. The trend for Long COVID publications (turquoise line) increased consistently, reaching 44.67% in 2023. The vertical dashed bright red line in 2021 represents the introduction of COVID-19 vaccinations

Following the widespread administration of COVID-19 vaccinations, there has been a notable and consistently growing increase in research focusing on long COVID [22] (Fig. 1).

Therefore, we dedicated a significant part of this work to contextualizing data synthesized from acute COVID-19 studies into long COVID research, discussing potential implications and rationales, with a focus on the RECLAIM study as a large-scale ongoing example.

## Methods

We observed the guidelines suggested by the PRISMA statement in conducting this systematic review and meta-analysis. The protocol was registered with PROSPERO, International Prospective Register of Systematic Reviews (ID = CRD42024513007).

## Search Strategy and eligibility criteria

The following databases were searched for publications that were available till 1 June 2024: PubMed/Medline, Web of Science, SCOPUS, CLINICALTRIALSGOV, CENTRAL/COCHRANE, ICTRP, Google Scholar, using the following query: (“COVID-19” OR “SARS-CoV-2” OR “novel coronavirus” OR "2019-nCoV “) AND (“Pentoxifylline” OR “Trental”). The search was updated on the 4th of July.

Studies were included in the systematic review according to the following eligibility criteria:

Study design: All clinical study designs, including clinical trials (RCTS and non-RCTS), observational studies, case reports, and case series were included.

Participants: Patients diagnosed with COVID-19, regardless of age, gender, severity of disease, or comorbidities.

Intervention(s): Pentoxifylline in any dosage or regimen, either alone or in combination with other treatments.

Comparator(s)/ control: Placebo, no treatment, standard care, or other pharmacological interventions.

Outcomes: Primary and secondary outcomes, including mortality, duration of hospitalization, inflammatory markers, and adverse events.

Non-human research studies, secondary research studies (reviews, systematic reviews, meta-analysis), and non-empirical studies (opinion pieces and hypotheses) were excluded.

## Study selection

After removing duplicates, two independent reviewers carried out the screening process blindly in two steps: Title and abstract screening, with excluding the ineligible studies. Full-text screening to verify the eligibility for the systematic review. In case of conflicts, they were resolved by discussion or consultation with a third author if necessary.

## Data extraction

**Two researchers independently extracted the data of included analytical studies,** using a standardized form designed for this systematic review.

The following key fields were included in the extended data extraction form: Study ID, study design, country, study groups, sample size, participants age and gender, COVID-19 details, dosage and regimen of pentoxifylline/ interventions, comparator, primary and secondary outcomes and outcome-measures, treatment duration, concise summary of study findings and conclusions.

## Risk of *bias* assessment

The risk of bias (ROB) appraisal was performed independently by two authors using tools specific to the study designs as follows:

Quality/ ROB assessment of clinical trials:

It was carried out using the Cochrane Collaboration's tool for randomized trials ROB2 for randomized trials and the ROBINSi tool for non-randomized studies. Assessments were conducted at the study level, focusing on domains such as the randomization process, deviations from intended interventions, missing outcome data, measurement of the outcome, and selection of the reported result.

## Data synthesis strategy

The results from the included studies were categorized based on predetermined outcomes such as mortality rates, length of hospital stay, and changes in inflammatory markers. Key findings from each study were summarized within each thematic area, highlighting study characteristics, pentoxifylline intervention details, and outcome measures. Assessments of study quality and risk of bias were integrated into the synthesis process, considering the strength of evidence when interpreting findings. Findings across studies within each thematic area were compared to identify consistencies, discrepancies, and trends, exploring potential sources of heterogeneity. The implications of synthesized findings for clinical practice and future research were discussed, emphasizing areas of consensus, remaining uncertainties, and implications for the use of pentoxifylline in COVID-19 patients. RevMan 5.3 software was used for data suitable for quantitative analysis. Changes in hospitalization days were pooled as Mean Difference (MD), and mortality rates as odds ratio, adopting a random effects model.

## Results

### Results of the Search

**Fig. 2:**
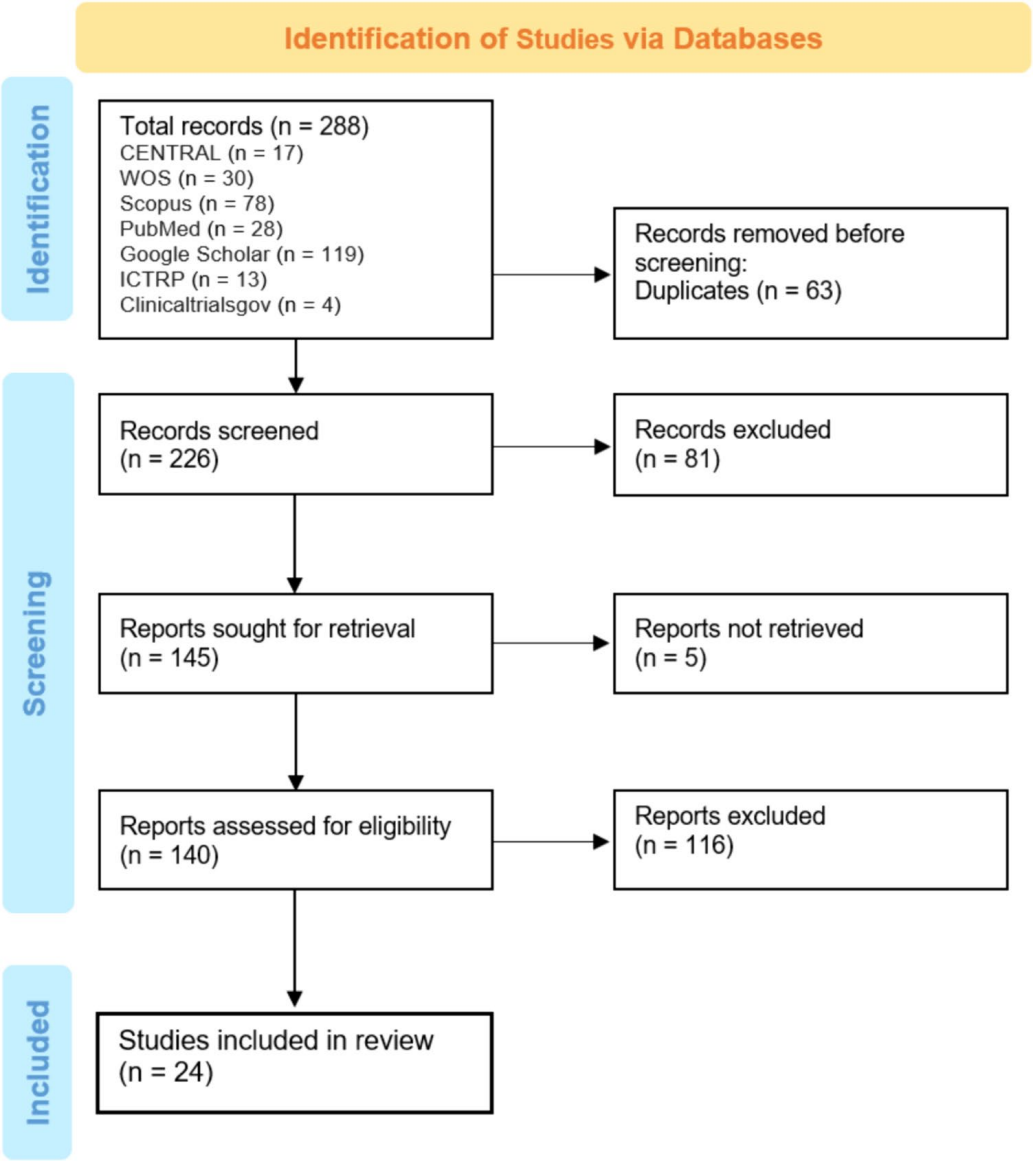
Study selection flow diagram

On June 1st, 288 studies were found across 7 electronic databases. The search was updated on the 4th of July and yielded the same results: Central (n = 17), Web of Science (n = 30), Scopus (n = 78), PubMed (n = 28), Google Scholar (n = 118), ICRTP (n = 13), and ClinicalTrials.gov (n = 4).After removing 63 duplicate studies, 225 articles remained for screening. From these, 139 studies were assessed for eligibility, with 24 meeting the criteria for inclusion in this systematic review. Studies that did not meet the specified inclusion criteria detailed in the methods section were excluded. The progression of this process is depicted in a flowchart (Fig. 2).

## Included studies

### A Compact List of brief IDs (author year) for the included studies

Azizi 2021 [23], Eghbali 2023 [24], Maldonado 2021 [25], Sarhan 2023 [26], Seirafianpour 2023 [27], Chavarría 2021 [28], Luévano 2022 [29] Wall 2020 [30], Cora 2021 [31], Chandrashekara 2020 [32], Eisa 2023 [33], Espinosa 2020 [34], Kaneria 2021 [35], Kler 2021 [36], 2021 Malinowski 2022 [37] Modrzejewska 2024 [38], Surya 2021 [39], Teixeira 2022 [40], Kelleni 2022 [41], Nassani 2021 [42], Topel 2021 [43], Toker 2023 [44], Valentim 2021 [45], Weltman 2021 [46].

## Risk of *bias* assessment

**Fig. 3:**
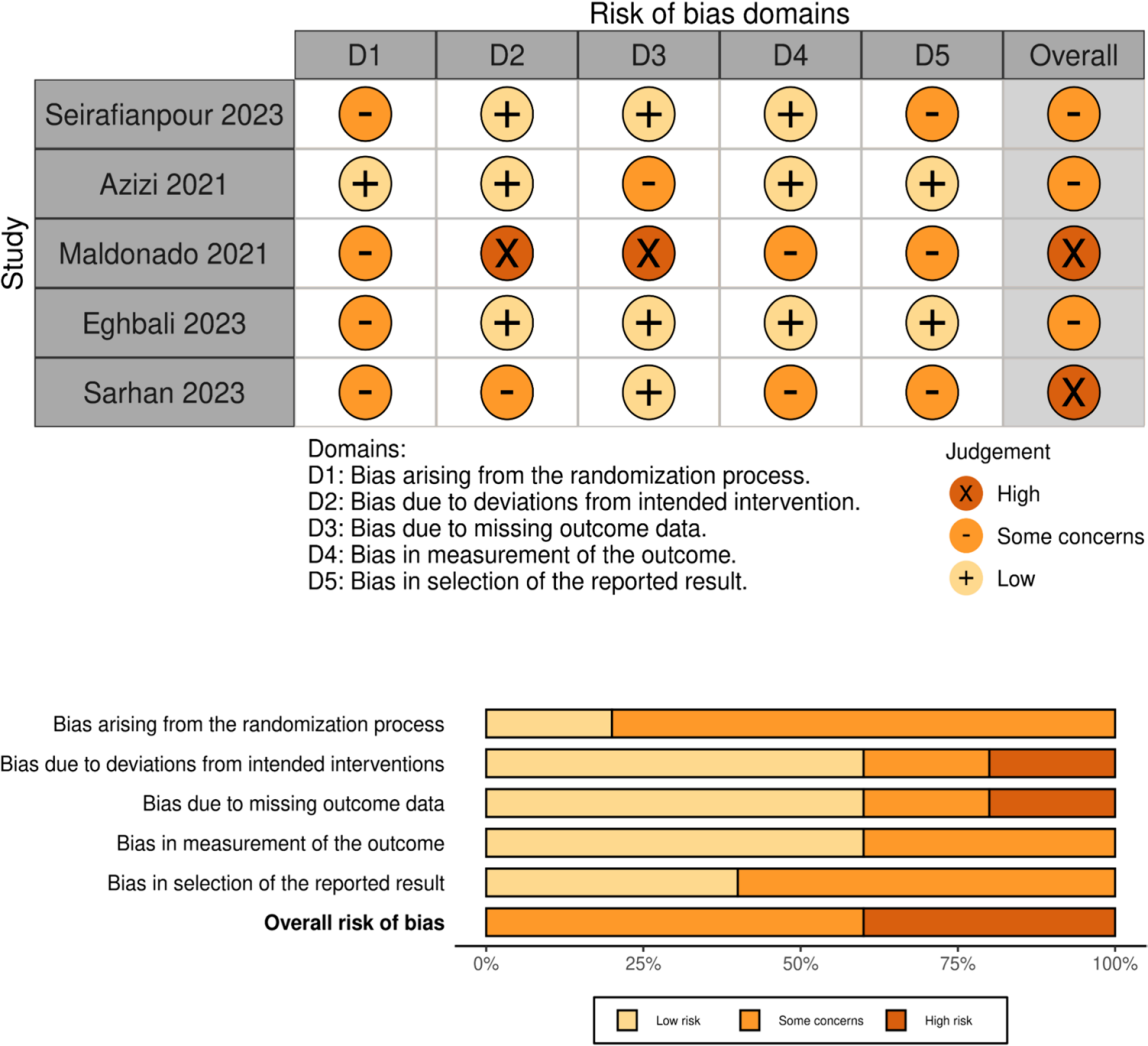
Risk of Bias Domains and Judgment for the included RCTs, using the ROB2 tool

Five RCT studies were assessed using ROB2, of which two studies had an overall “high” risk of bias, and the remaining three studies had an overall “some concerns” risk of bias, as illustrated in Fig. 3.

The rest were evaluated using relevant risk of bias assessment tools provided in the supplementary material.

## Description of studies

Regarding the design of the included studies, five were RCTs with a total sample size of 448 patients, two were non-randomized trials with 161 patients, and one was a retrospective cohort study with 209 patients, along with seven case reports and nine case series. The analytical studies provided clinical data on a total of 818 COVID-19 patients, of whom 425 received PTX in addition to their standard therapy. The details regarding the summary and characteristics of these studies and patients are presented concisely in Table 1 and in an extended form in the supplementary material.

**Table 1:** Summary of characteristics of the included analytical studies

Study	Design	Sample size	Intervention	Comparator	Treatment duration (days)	Conclusion
Eghbali 2023	RCT Double-blind	Total: **120** Intervention: 60 Control: 60	PTX (400 mg every 12 h) and colchicine (0.5 mg daily), in addition to standard therapy	Standard therapy	Duration of hospital stay	Compared with standard therapy alone, the co-administration of PTX and colchicine significantly reduced the duration of fever and length of hospital and ICU stays The two groups had no significant difference in mortality, clinical and laboratory results
Sarhan 2023	RCT Open-label	Total: **68** Intervention: 30 Control: 38	PTX (400 mg three times daily, orally) in addition to standard therapy	Standard therapy	7	PTX did not exert significant improvements in clinical outcomes, compared with standard therapy alone PTX exerted a favorable impact on CRP and IL-6, with no significant adverse effects
Seirafian Pour 2023	RCT Double-blind	Total: **150** Intervention: 50 Control: 100	PTX (400 mg three times daily, orally) in addition to standard therapy	Standard therapy	14	PTX significantly reduced the mortality rate and length of hospital stay, compared with standard treatment alone PTX also showed a favorable impact on inflammatory biomarkers (CRP, LDH, ESR)
Maldonado 2021	RCT Open-label (pilot study)	Total: **38** Intervention: 26 Control: 12	PTX (400 mg three times daily, orally) in addition to standard therapy	Standard therapy	Duration of hospital stay	PTX treatment was associated with increased lymphocyte count and decreased LDH levels, although no difference was observed regarding days of hospitalization, mortality, and need for intubation
Azizi 2021	RCT Double-blind	Total: **72** Intervention: 40 Control: 32	PTX (400 mg three times daily, orally) in addition to standard therapy	Placebo in addition to standard therapy	14	PTX did not show any superiority over placebo in improving the clinical outcomes, although it had a beneficial effect on IL-6 and showed an acceptable safety profile
Chavarría 2021	Non-randomized clinical trial	Total: **110** Moderate: 59 Severe: 51	-PTX: 400 mg every 12 h, orally or by nasogastric tube -Antioxidants: every 12 h, orally or by nasogastric tube	Baseline	5	Antioxidant therapy + PTX improved the survival scores, oxidative, and inflammatory markers PTX only did not show significant changes in oxidative markers, but there was an improvement in inflammatory markers
Wall 2020	Retrospective cohort	Total: **209** Intervention: 58 Control: 151	Patients with a history of asthma or chronic obstructive pulmonary disease were given theophylline, all other patients received PTX (400 mg three times daily, orally)	Standard therapy	7	PTX and theophylline were associated with an increase in ROX score, a decrease in CRP and mortality, with few adverse events
Luévano 2022	Trial (Pilot study)	Total: **51**	PTX (400 mg) in addition to supportive measures	Baseline	28	PTX, in addition to supportive measures, results in 100% survival in patients with SARS-CoV-2 and chronic liver disease as well as avoidance of admission to the ICU

*PTX*: pentoxifylline, *ICU*: intensive care unit, *CRP*: C-reactive protein, *LDH*: lactate dehydrogenase, *IL-6*: interleukin-6

## Narrative summary of patterns and findings across studies

In the included analytical studies, PTX was administered as an adjuvant therapy to the standard COVID-19 regimen in one group and compared to the control group receiving the standard therapy alone, except for two studies that designed no comparison with the standard protocol, namely Chavarría 2021 and Luevano 2022. The dose was 400 mg, three times daily in most studies (Sarhan 2023, Seirafianpour 2023, Maldonado 2021, Azizi 2021, Wall 2020) and twice daily in two studies (Eghbali 2023, Chavarría 2021). Treatment duration roughly ranged from one to two weeks.

The reported aim of using PTX as an adjuvant to the standard COVID-19 protocol was to test its potential to improve clinical outcomes and mitigate the excessive inflammatory response caused by COVID-19, thereby enhancing patient survival and reducing the duration of hospitalization and mortality rates.

Here we aim to present a concise and useful summary of PTX's effects on various clinical and laboratory measures among COVID-19 patients in those studies.

Most of the studies reviewed suggest that adding pentoxifylline to standard therapy may contribute to a shorter hospital stay for patients compared to the standard protocol alone, which was a stated primary outcome in most of the studies that reported it. However, the degree of significance varies across the studies.

Mortality rates among patients treated with pentoxifylline were reported in most studies as well. Their findings indicate mixed results regarding the efficacy of pentoxifylline add-on in reducing mortality rates among the studied COVID-19 patients. While a Large-scale study reported a stark reduction in mortality (Seirafianpour 2023), several others did not find statistically significant differences (Maldonado 2021, Sarhan 2023, Azizi 2021, Eghbali 2023, Wall 2020).

Worth mentioning, that the study with the longest follow-up period (Luevano 2022) had a mortality rate of zero percent. However, its methodology does not appear to be that rigorous.

**Fig. 4:**
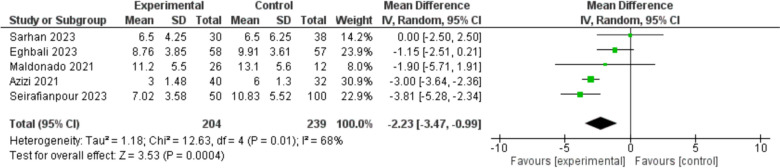
Duration of Hospitalization: Analyzing data from 5 studies with 443 participants, there was a significant reduction in hospitalization duration for the experimental group compared to the control group. The pooled Mean Difference (MD) was −2.23 days, with a 95% Confidence Interval (CI) of −3.47 to −0.99, indicating a statistically significant reduction (p = 0.0004). Heterogeneity was moderate (I.^2^ = 68%)

**Fig. 5:**
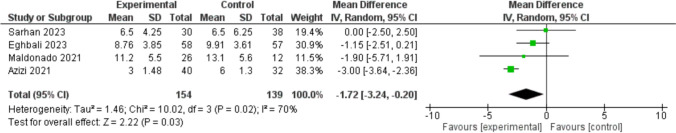
Duration of Hospitalization (Sensitivity Analysis): A subsequent analysis of data from 4 studies with 293 participants, after excluding the study with the greatest influence, still demonstrated a significant reduction in hospitalization duration for the experimental group compared to the control group. The pooled Mean Difference (MD) was −1.72 days, with a 95% Confidence Interval (CI) of −3.24 to −0.20, indicating a statistically significant reduction (p = 0.03). Heterogeneity was moderate (I^2^ = 70%)

**Fig. 6:**
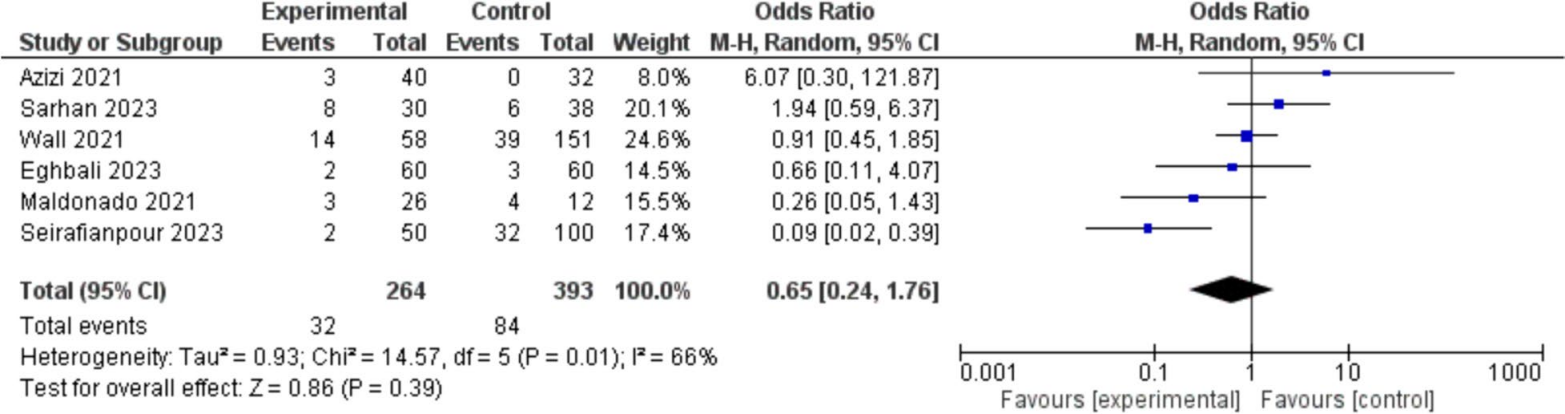
Mortality: Analysis of 6 studies with 657 participants, showed no significant difference in mortality between the experimental and control groups. The pooled Odds Ratio (OR) was 0.65, with a 95% Confidence Interval (CI) of 0.24 to 1.76, indicating no statistically significant reduction (p = 0.39). Heterogeneity was moderate (I^2^ = 66%)

The two aforementioned important and relatively well-reported outcomes, hospitalization duration and mortality rate are further explored through the statistical synthesis in the meta-analysis section in Figs. 4, 5, and 6.

Regarding ICU, patients in the Sarhan 2023 study were all in the ICU at the beginning of that study. In Wall 2020, around 60% of patients in both groups were already on mechanical ventilation at baseline, indicating intensive care. All other reported studies include patients with moderate to severe disease status. Two studies focused on and reported ICU-admission-related outcomes. Eghbali 2023 demonstrated statistically significant reductions in both ICU admissions and the length of ICU stay for the pentoxifylline group, while Azizi 2021 did not. Additionally, the longest study, Luevano 2022, reported zero percent need for ICU admission over 28 days.

In apparent accordance with its hypothesized effects, pentoxifylline influenced several key inflammatory biomarkers.

Almost all groups and subgroups in Chavarria exhibited statistically significant reductions in the proinflammatory markers CRP and IL-6. In intergroup comparisons for CRP, the intervention group in Seirafianpour demonstrated a notable reduction, whereas no such effect was observed in Azizi and Sarhan. Regarding IL-6, Azizi showed a significant intergroup effect favoring the experimental group, while Sarhan did not exhibit a similar trend. The effect of PTX on LDH was statistically significant compared to the control group in Seirafianpour and Maldonado but not in Sarhan.

Adverse events related to PTX were generally not significant across the analytical studies. No major adverse reactions directly attributable to PTX were reported.

## *Meta*-analysis

### Discussion

This work intends to offer a systematic review of clinical studies and a meta-analysis, aiming to evaluate the effects of including Pentoxifylline (PTX) in the treatment of acute COVID-19, covering its influence on several clinical outcomes and inflammatory markers, including length of hospital stay, mortality rate, ICU admission, adverse events, CRP, IL-6, and LDH and could be considered the first one assessing empirical data on the topic. Looking back, PTX was proposed for use during the SARS-CoV-1 outbreak in 2003, but it was not clinically studied, likely due to the rapid resolution of the epidemic [47, 48].

## Summary of key findings

Twenty-four clinical studies, including 8 analytical studies involving 818 COVID-19 patients, of whom 425 received pentoxifylline (PTX), were included.

Hospitalization duration was reduced in most studies with the addition of PTX, and the meta-analysis showed a significant pooled effect. A subsequent sensitivity analysis confirmed the significant reduction in hospitalization duration, even after excluding the most influential study.

Mortality rate findings were varied. Seirafianpour 2023 observed a notable reduction in mortality; however, baseline comorbidity differences between the two groups were significant to start with. On the other hand, studies by Maldonado 2021 and Azizi 2021 found no significant differences in mortality, as there were no initial significant differences between the two groups at baseline, making their results probably more indicative. Indeed, the meta-analysis indicated no significant difference in mortality rates, suggesting that the effect of PTX on mortality remains inconclusive. This may be due to the large percentage of patients with serious comorbidities and the short study durations.

Inflammatory Biomarkers: In Chavarria, most groups and subgroups showed significant reductions in CRP and IL-6. Seirafianpour's intervention group showed a notable reduction in CRP, unlike Azizi and Sarhan. For IL-6, Azizi showed a significant effect in the experimental group, unlike Sarhan. PTX significantly affected LDH in Seirafianpour and Maldonado but not in Sarhan.

Standard therapy across the different studies, even within the same country, was not that standard, which may have been a major cause of heterogeneity in various outcomes.

ICU-Related Outcomes: Eghbali 2023 showed significant reductions in ICU admissions and ICU stay length with PTX, while Azizi 2021 did not. Luevano 2022 reported zero ICU admissions over 28 days.

Safety: PTX was well-tolerated with no significant adverse events reported.

## Long COVID

Neuropsychiatric, neurological, cardiopulmonary, gastrointestinal, or functional mobility symptoms that persist, return, or emerge weeks to months after the acute phase of SARS-CoV-2 infection and may last for weeks, months, or longer, often occurring in clusters and not explained by alternative diagnoses, are referred to as post-acute COVID-19 syndrome (PACS), Post-Acute Sequelae, or simply Long COVID​ [49, 50].

Neuropsychiatric symptoms of long COVID include fatigue at 29.2%, cognitive impairment or brain fog at 28.85%, anxiety at 27.77%, depression at 22.44%, sleep disturbances at 19.13%, and headaches at 18.05%. Other symptoms, while still significant, are less frequent compared to neuropsychiatric symptoms (NPS) [51].

Many of these neuropsychiatric symptoms could potentially be positively affected by pentoxifylline, and it has indeed been evaluated in various clinical trials involving affective, psychotic, cognitive, neurotic, and behavioral conditions, showing promising results in a significant number of these studies. [52–60].

To elaborate on the affective aspect as an example, four randomized, double-blind, placebo-controlled trials that used PTX versus placebo for treating depressive symptoms in MDD showed significant differences in depressive symptoms. In three of the four trials, PTX was added to standard therapy as an adjuvant, which may be considered a limitation of the treatment approach. However, the one study that used PTX as a sole intervention also reported a significant reduction in depressive symptoms compared to placebo, but—unlike the other studies—it did not achieve a significant difference in response rates. None of the studies showed a significant difference between PTX and placebo in terms of side effects [52–55].

However, several studies on PTX for NPS did not show significant improvements, and none specifically addressed long COVID. The lack of specific research on long COVID, the fact that many studies used PTX as an adjuvant therapy rather than as a sole intervention, and the administration schedule of PTX, which may impact patient adherence over extended periods, must be considered when evaluating its potential use in long COVID management.

Prolonged hospitalization duration has been identified as a risk factor for the development of long COVID, and our study shows that the use of pentoxifylline as an add-on to the COVID regimen significantly reduces hospitalization duration, potentially reducing the risk of long COVID [61–63].

This could be seen as yet another rationale to explore PTX further in that context.

Recovering from COVID-19 Lingering Symptoms Adaptive Integrative Medicine Trial (RECLAIM: NCT05513560) is a Canada-wide phase II/III adaptive, partially triple-blind, randomized controlled platform trial studying interventions for long COVID. Participants (N = 1000) with symptoms lasting beyond 3 months are randomized to PTX (400 mg pill 3 times/day), Ibudilast, or placebo, with a treatment duration of 2 months and follow-up for 6 months, with an estimated primary completion date in May 2025. Outcome measures shall assess comparative PTX effects on functional limitations, Neuropsychiatric symptoms (Fatigue, Anxiety, Depression, Cognitive difficulties, Brain fog, Insomnia), Shortness of breath/ Dyspnea, Tachycardia, and Gastrointestinal issues.

## Long-standing literature limitations and future research recommendations

The present attempt for meta-analysis was limited by the deficient reporting of continuous variables in many of the included studies, which prevented their use in statistical synthesis without substantial imputations with questionable accuracies.

In scientific research in general, this remains a common defect that often brings to mind the broader, non-overseeable dilemma of data transparency in clinical research. We hope to encounter less of this in the future.

## Conclusion

The systematic review and meta-analysis indicate that the addition of pentoxifylline (PTX) to standard COVID-19 therapy may offer certain clinical benefits, particularly in reducing the duration of hospitalization. Pooled data suggest a statistically significant reduction in hospitalization duration for patients with moderate to severe COVID-19 receiving PTX. Such consistent and in multiple studies notable reductions in hospitalization duration might imply a faster recovery and could indicate a risk reduction of later developing long COVID.

In apparent accordance with its hypothesized effects, pentoxifylline frequently improved inflammatory markers such as CRP and IL-6 in some studies but not uniformly; however, the impact of PTX on mortality rates among COVID-19 patients remains inconclusive, with no significant reduction observed in the pooled analysis within the relatively short timeframes of the studies.

Adverse events related to PTX were generally minimal, with no significant safety concerns reported.

Findings from these acute COVID-19 treatment studies may provide insights into PTX's potential utility for reducing the risk of post-acute COVID-19 syndrome and managing symptoms associated with long COVID.

## Supplementary Information

Below is the link to the electronic supplementary material.

Supplementary file1 (XLSX 457 KB)

## Data Availability

No datasets were generated or analysed during the current study.
